# Virtual Sensor for Failure Detection, Identification and Recovery in the Transition Phase of a Morphing Aircraft

**DOI:** 10.3390/s100302188

**Published:** 2010-03-17

**Authors:** Guillermo Heredia, Aníbal Ollero

**Affiliations:** 1 Robotics, Vision and Control Group, University of Seville, Camino de los Descubrimientos s/n, 41092, Seville, Spain; 2 Aerospace Technologies Advanced Center (CATEC), Aeropolis, 41309, Seville, Spain; E-Mail: aollero@cartuja.us.es

**Keywords:** virtual sensors, UAVs, fault detection, identification and recovery, morphing aircraft

## Abstract

The Helicopter Adaptive Aircraft (HADA) is a morphing aircraft which is able to take-off as a helicopter and, when in forward flight, unfold the wings that are hidden under the fuselage, and transfer the power from the main rotor to a propeller, thus morphing from a helicopter to an airplane. In this process, the reliable folding and unfolding of the wings is critical, since a failure may determine the ability to perform a mission, and may even be catastrophic. This paper proposes a virtual sensor based Fault Detection, Identification and Recovery (FDIR) system to increase the reliability of the HADA aircraft. The virtual sensor is able to capture the nonlinear interaction between the folding/unfolding wings aerodynamics and the HADA airframe using the navigation sensor measurements. The proposed FDIR system has been validated using a simulation model of the HADA aircraft, which includes real phenomena as sensor noise and sampling characteristics and turbulence and wind perturbations.

## 1. Introduction

Reconfigurable or morphing aircraft are flight vehicles that change their shape to effect both a change in their mission and to perform flight control without the use of conventional control surfaces [1]. The aircraft with morphing capability provides the advantage of being able to fly multiple missions, which is not possible by those based on the conventional aircraft designs.

In the last 60 years there have been many attempts to design a reconfigurable vehicle that could join the VTOL (Vertical Take-Off and Landing) and hovering capabilities of helicopters and the cruise speed and efficiency of airplanes, as the tilt-rotor concept [2], the compound helicopter [3] or the XV1 convertiplane [4].

**Figure 1.**
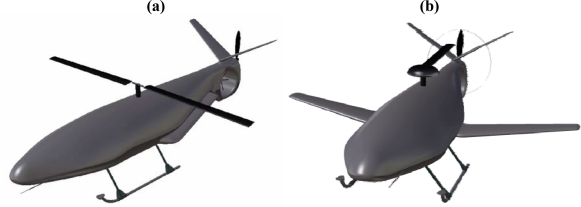
HADA conceptual design. (a) Helicopter configuration. (b) Airplane configuration.

The “Helicopter ADaptive Aircraft” (HADA) is a project under development since 2007 by a consortium of 26 Spanish companies, universities and research institutions, led by the National Institute for Aerospace Technology (INTA). The objective of the HADA [5,6] project is the development of a reconfigurable Unmanned Aerial Vehicle (UAV) that performs as a helicopter for take-off, landing and hovering flight (Figure 1a), but that “morphs” to a conventional fixed wing configuration (Figure 1b) for cruise flight. The innovative design of the HADA concept relies on that it reconfigures itself in flight by deploying the two half-span wings which are hidden beneath and along the fuselage while in helicopter mode. The morphing process is completed by transferring the engine power to a pusher propeller at the rear end of the fuselage, stopping the rotors and holding backwards the blades of the main rotor. The process is reversed when morphing to helicopter flight mode.

The HADA project envisages the development of three prototype UAVs. The first one is the *Colibri* (the Spanish word for hummingbird), a reduced scale model for aerodynamic wind tunnel testing and flight testing. Several instances of the Colibri have been developed since 2007 for different aerodynamic tests in airplane, helicopter and transition modes. The second prototype is the *Alondra* (Spanish word for lark), a 2/3 scale UAV demonstrator with all the relevant mechanisms and functions of the HADA concept, which is in development and will be available in 2010. The third is known as *Libelula* (Spanish word for dragonfly), the full scale HADA prototype which is also in development. The preliminary design for the Libelula UAV system includes a main rotor diameter of 6 m, a wing span of 6 m and a wing area of 4 m^2^. The mass is 380 kg, the required power is 130 kW and the transition speed is approximately 50 m/s.

A typical HADA mission may have the following phases:

1. Take-off in helicopter mode with the wings folded under the fuselage; flight control as a conventional helicopter.
2. When flying forward at the transition speed, gradually unfold the wings while controlling the aircraft with helicopter controls.
3. Once the wings are unfolded, begin power transition between main and tail rotor and the propeller.
4. When full power is transferred to the propeller, fold main rotor and continue flying in aircraft mode with aircraft controls.

Once the targeted area has been reached, the inverse process can be performed and the HADA can operate in helicopter mode to fulfill the mission.

Operational reliability and safety of HADA is extremely important, and a health monitoring and condition-based maintenance system for it is being developed [6]. Being a morphing UAV, new failure modes may appear during the reconfiguration process, as for example sensor and actuator failures in morphing surfaces and failures in power transmission mechanisms. Since one of the main HADA morphing processes is the folding and unfolding of the wings, special efforts have to be devoted to assure reliability of sensors and actuators in this transition phase. This paper concentrates on detection of faults in the wing deployment sensors and actuators.

Reliability has always been a main issue in UAVs [7], where Fault Detection, Identification and Recovery (FDIR) techniques play an important role in the efforts to increase the reliability of the systems. Most FDIR applications to UAVs that appear in the literature use model-based methods, which try to diagnose faults using the redundancy of some mathematical description of the system dynamics. FDIR has been applied to unmanned aircraft, either fixed wing UAVs [8] or helicopter UAVs [9–11]. However, in most cases FDIR has been applied to navigation sensors and actuators, and not to sensors and actuators used in aircraft internal reconfiguration. Furthermore, wing deployment changes significantly the aerodynamics of the aircraft as well as the inertia and mass distribution, being a nonlinear dynamic process.

Virtual Sensors [12] (also known as soft sensors) are software modules which utilize measurable signals (Virtual Sensor inputs) in order to reconstruct a signal of interest (Virtual Sensor output). Virtual Sensors are useful in replacing physical sensors, thus reducing hardware redundancy and acquisition cost, or as part of fault detection methodologies by having their output “compared” to that of a corresponding actual sensor.

Although Virtual Sensors may be developed based upon mathematical models obtained directly from the physics of the system and first principles, in many cases such mathematical models are unavailable, or their exact parameter values are unknown, or they are too complicated to be used. For this reason the development of Virtual Sensors often has to be based upon system identification techniques.

There exist in the literature some works that propose nonlinear Virtual Sensors in aerospace applications [12–14], although they have primarily been applied to navigation sensors and actuators. In this paper, a nonlinear virtual sensor is proposed to estimate the sweeping angle of the wing. The virtual sensor is obtained using Nonlinear AutoRegressive with eXogenous excitation (NARX) system identification.

The paper is organized as follows. Section 2 presents an overview of the model of the HADA aircraft. Section 3 describes the structure of the proposed virtual sensor based FDIR system. Section 4 presents some simulation experiments to illustrate the behavior of the virtual sensor in case of sensor and actuator failures. Finally, the conclusions in Section 5 complete the paper.

## 2. Overview of the HADA Model

At the present development stage of the HADA project it is not possible to make flight tests which include wing folding or unfolding, and therefore the proposed virtual sensor-based FDI system cannot be tested in flight. Thus, a HADA model and simulator have been developed for that purpose, and also for controller development. Furthermore, there are available several wind tunnel tests with variable sweep wings with one of the prototypes, and they have been used to validate HADA aerodynamics modeling.

The HADA aircraft can be modeled as a common airplane or helicopter when it is operating either in airplane or helicopter modes, and the motion equations can be found in many textbooks (see, for example [15,16]). Aerodynamic forces and moments are incorporated in the equations of motion in the form of *aerodynamic stability and control derivatives*. Stability and control derivatives are measures of how particular forces and moments on an aircraft change as other flight variables change, and are based on linear Taylor series expansions to define the aerodynamics of the aircraft [15]. The linear aerodynamic model is based on small perturbation control theory around a single flight condition, which for this work is the transition of HADA between airplane and helicopter modes.

On the other hand, it is not so easy to model HADA aerodynamics in the transition phases when the wings are folding or unfolding. Mode transition in the HADA aircraft will take place at velocities around 40–50 m/s. The velocity of the wing tip in the folding/unfolding movement of the wing is very low compared to the airflow speed around the wing. Thus, it is reasonable to assume that it is a quasi-steady process, and the aerodynamic stability and control derivatives approach can be used.

The Vortex Lattice Method (VLM) is a numerical method built on the theory of potential flow, which has been widely used for aircraft design [17–18]. The method is based on the idea of a vortex singularity as the solution of Laplace’s equation. The VLM models the lifting surfaces of an aircraft as an infinitely thin sheet of discrete vortices to compute lift and induced drag. The Tornado program [19] has been used as the VLM code in this paper. Tornado is able to calculate the lift and drag forces and also the stability and control derivatives.

Modelling of the HADA for different wing sweep angles has been done in the following way: for each value of the sweep angle the effective airfoil (the wing section that the main airflow “sees”) has been obtained as the result of an oblique section of the wing. Then, the VLM has been used to obtain the stability and control derivatives corresponding to this wing sweep angle. This set of coefficients has been integrated in the HADA simulation model, which has been developed using Matlab/Simulink. A detailed description of the model can be found in [20].

The actuation mechanism for wing folding and unfolding is an electric linear actuator linked to the two halfwings which will rotate around pivot points. The wing actuator has been modelled as a first order system with limited angular deflection and maximum angular rate.

The HADA navigation system provides to other subsystems the position, velocity and attitude information they need to control the aircraft, manage the mission or inform the remote pilot. The navigation sensor pack that will be installed on the HADA prototypes will typically include an Inertial Measurement Unit (IMU), which provides data from three accelerometers, three gyroscopes and a 3-axis magnetometer, a Global Positioning System (GPS) receiver which gives absolute position and also absolute velocity estimations, absolute and differential pressure sensors, which are used to measure aircraft altitude and total velocity with respect to the air mass, and a nose probe providing measurements for the angle of attack and the sideslip angle. The measurements of these navigation sensors are integrated by the navigation system to obtain the aircraft state using a Kalman filter [21].

Although all these sensor measurements are combined to obtain an estimation of the HADA position and orientation angles, the FDIR virtual sensor uses the sensor readings directly to exploit all the available information.

The HADA aircraft will include also a Hall effect rotary position sensor to measure directly the wing sweeping angle, although it may not be present in one of the prototypes. For this reason, the virtual sensor FDIR system has been developed considering both possibilities, that the wing sweeping angle sensor is available or not. All these sensors have been modelled in the simulator, including noise characteristics.

## 3. Virtual Sensor for FDIR

### 3.1. FDIR Structure

The monitoring of faults in feedback control system components is known as Fault Detection, Identification and Recovery (FDIR), which is composed of three main functions: the process of determining that a fault has occurred (detection), the localization of the fault within the system (identification) and the process of limiting the fault propagation and enabling the service to be restored to an acceptable state (recovery). The procedure of generating a control action which has a low dependency on the presence of certain faults is known as fault tolerant control.

**Figure 2.**
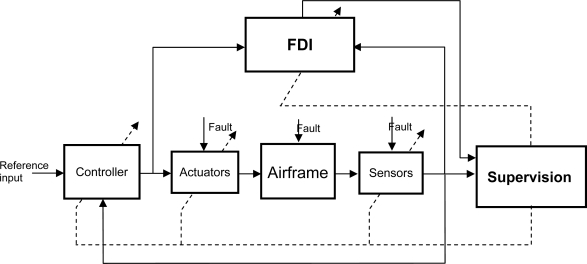
FDIR general structure.

Figure 2 shows the general schematic arrangement appropriate to many fault tolerant control systems [22] with four main components: the plant itself (including sensors and actuators), the Fault Detection and Identification (FDI) unit, the feedback controller and the supervision system. The plant is considered to have potential faults in sensors, actuators or other components. The FDI unit provides the supervision system with information about the onset, location and severity of any fault. Based on system inputs and outputs together with fault decision information from the FDI unit, the supervision system will reconfigure the sensor set and/or actuators to isolate the faults, and tune or adapt the controller to accommodate the fault effects. The FDI and Supervisor blocks in Figure 2 jointly perform the FDIR functions.

**Figure 3.**
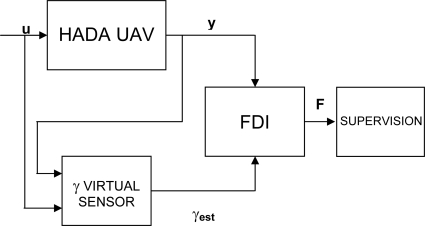
Virtual sensor based FDIR.

The scheme proposed in this paper for FDIR on sensors and actuators of the wing deployment process is presented in Figure 3, where **u** and **y** are the inputs and outputs vectors of the HADA UAV, respectively. The virtual sensor block implements the wing sweeping angle virtual sensor. The output of this block is an estimation of the sweeping angle, γ_est_. The FDI block contains the logic for fault detection and identification, and generates a fault signal **F**, which is used by the supervision unit to decide if a reconfiguration of HADA sensors, actuators and controller is necessary to recover from a declared fault.

The virtual sensor takes as inputs the HADA inputs **u** and outputs **y**, and reconstructs the sweeping angle γ from these signals. The wing sweeping angle is an internal variable which does not participate directly in the HADA dynamics. The relationship is indirect: as the wings are unfolding, the lift and drag forces generated by the wing interact with the airframe, and this interaction is reflected in the inputs and outputs of the HADA. This relationship will be nonlinear, and therefore it is not possible to use standard linear input-output models. Thus, a nonlinear input-output model has been used, which is described in the next section.

### 3.2. Nonlinear Input-Output Model for Wing Sweeping Angle Virtual Sensor

In order to account for the nonlinear dynamical relationships between the signal under reconstruction (virtual sensor output, the wing sweeping angle) and the measurable signals used for this purpose (virtual sensor inputs, the inputs and outputs of the HADA aircraft), a stochastic Multiple-Input Single-Output (MISO), Nonlinear AutoRegressive with eXogenous excitation (NARX) [23] model structure of polynomial form is adopted for virtual sensor implementation.

Consider a general discrete time nonlinear system with one output *y* and *m* inputs *u*_1_,…,*u_m_*. The well-known linear AutoRegressive with eXogenous inputs (ARX) [23] model supposes that the current output *y*(*t*) is predicted as a weighted sum of past output values and current and past input values (known as regressors). The NARX structure is an extension of the linear ARX structure: instead of the weighted sum that represents a linear mapping, the NARX model has a much more flexible nonlinear mapping function:

(1) $$y(t)=f\;(y(t-1),\ldots ,\;y(t-n_{y}),\;u_{1}(t-1),\ldots ,\;u_{1}(t-n_{u1}),\ldots ,\;u_{m}\;(t-1),\ldots ,\;u_{m}\;(t-n_{\mathrm{um}}))$$

where *n_y_* and *n_ui_*, *i* = 1,2,…,*m,* denote the maximum lags in the output and inputs, and *f*(·) is a suitable nonlinear function, a priori unknown. If *f*(·) is approximated as a polynomial of degree *M*, model (1) can be written as:

(2) $$y(t)=\sum_{i=0}^{r}\beta_{i}\;x_{i}(t)$$

where *r* depends on *m*, *M*, *n_y_*, and *n_ui_*, *i*=1,2,…,*m.* The parameters *β_i_*, *i* = 1,2,…,*r*, are suitable coefficients to be identified, *x*_0_ = 1 and *x_i_*(*t*), *i* = 1,2,…,*r*, are monomials made up of delayed outputs and/or inputs. For instance, if *n_y_*, = *m* = *n_u_* = 1 and *M* = 2, the most general model (2) (with positive powers) would be:

(3) $$y(t)=\beta_{0}+\beta_{1}y(t-1)+\beta_{2}y{\left(t-1\right)}^{2}+\beta_{3}u(t-1)+\beta_{4}u{\left(t-1\right)}^{2}+\beta_{5}y(t-1)u(t-1)$$

In the following, *β* = [*β*_0_ *β*_1_…*β_r_*] is the vector of unknown parameters, and:

(4) $$\varepsilon (t)=y(t)-\sum_{i=0}^{r}\beta_{i}\;x_{i}(t)$$

denotes the residual sequence. Assuming that *N* observations of the input and output variables are available, the least-squares (LS) estimate *β^LS^* of *β* is:

(5) $$\beta^{\mathrm{LS}}=\text{arg}\;\underset{\beta }{\text{min}}\sum_{t=1}^{N}{\varepsilon (t)}^{2}$$

If the nonlinear model structure (2) is assigned, that is the number and the form of the regressors *x_i_* in (2) are specified, the estimation problem is easily solved by means of standard algorithms. Since this NARX model formulation is linear in the parameters, standard LS algorithms can be used to estimate the parameters *β^LS^*. The NARX models are chosen with the structure that achieve the smallest Akaike’s Information Theoretic Criterion (AIC) [24], according to a simple search algorithm, in which the first half of data is used for estimation and the second for cross validation.

### 3.3. FDIR Logic

The FDI block has three signals available for detection and identification of faults in sensors and actuators of the wing folding/unfolding mechanism. The first one is the actual sweeping angle sensor signal (GSENS) if it is available, the second one is the estimation of the sweeping angle reconstructed by the virtual sensor using measurements of other sensors (GVIRT), and the third one is generated from the actuator command, modeling the actuator as a first order system (GACT).

For fault detection, if the sweeping angle sensor is available it is sufficient to compare the signals GSENS and GACT and compute a residual between them. In fact, this is possible also without implementing the virtual sensor. However, if only these two signals are available, it is impossible to isolate the fault to determine if it is located in the actuator or in the sensor. The residuals generated from the comparison of the virtual sensor output GVIRT with GSENS and GACT give independent additional information on the presence of a fault. In case the sweeping angle sensor is not available, only the residual of GVIRT and GACT can be used.

The residuals *R_i_* in this case are defined as the difference of the signals. Ideally, if no fault is present, the residual would be zero. In practice, the residual will take non-zero values due to estimation errors, sensor noise, perturbations, *etc.* Usually, the residual for a specific sensor will be bounded, and therefore a “threshold level” can be defined so that the absolute value of the residual is always below it in the absence of failures. The first time the residual goes above the threshold level, the fault is assumed to be present.

The fault detection procedure is designed to decide if the observed changes in the residual signal *R_i_* can be justified in terms of the disturbance (measurement noise) and/or modelling uncertainty as opposed to failures. It is critical to minimize the detection delay associated with a ‘true’ fault; furthermore, the false alarm rate should be minimized while, at the same time, no ‘true’ faults should remain undetected.

A well-known filter for the detection of moderate persistent shift in the mean value of the residual is the cumulative sum (CUSUM) filter [25]. This filter is used to detect both positive and negative changes in the mean value of the residual *R_i_* caused by the occurrence of a fault. The CUSUM filter with forgetting factor will be used to detect changes in the mean of the residuals of the wing sweeping angle.

Once a fault has been detected, it is very important to isolate the location of the fault. If there is no sweeping angle sensor installed it is obvious that the fault will be located at the wing actuator. But in case there is an angle sensor, it is critical to know if the fault is located in the sensor or in the actuator, because the fault location will determine the actions taken by the supervisor. This is impossible to do if the virtual sensor is not implemented. But if the three signals explained above are available, a simple fault identification logic can be defined. In brief, if GSENS is similar to GVIRT, and GACT differs from the other two signals, it means that the angle sensor is working properly and the fault is in the actuator, which is receiving a command that it is not able to execute. On the other hand, if GVIRT is different from GSENS, and it is similar to GACT, it means that the actuator is working properly, and it is the angle sensor that is faulty.

The fault identification that is provided by the virtual sensor is very important for the supervision unit. If the fault is in the sensor, the supervisor may decide to continue with the mission (maybe with degraded performance), substituting the faulty angle sensor signal with the angle virtual sensor signal. However, if it is the actuator that is faulty, it will probably decide to abort the mission, return to base and perform an emergency landing (depending on the wing sweeping angle that the actuator has stuck).

## 4. Simulation Experiments

Many simulation experiments have been performed with the HADA simulator presented in Section 2 to test the virtual sensor based FDIR system. The simulations have been done trying to reconstruct real flight conditions, and therefore sensor noise and perturbations have been taken into account. Sensor characteristics have been considered modeling their sampling frequency and additive Gaussian white noise. The main sources of perturbations in aircraft flight are air turbulence and wind gusts. Moreover, it is important to note that the mechanism which lets the virtual sensor act works in the following way: as the wings are unfolding, lift and drag forces are produced in the wing, and these forces and moments are transferred to the aircraft. As a consequence, flight variables (velocities, orientation angles, *etc.*) change, and the control system acts changing the actuator commands to maintain level flight. The virtual sensor described in this paper models the relationship between the flight variables and actuators (which are available to the FDIR system through the sensors) and the wing sweeping angle. It is clear that wind turbulence will modify these relationships, and therefore it will introduce errors in the virtual sensor estimate.

In this section, several simulation experiments are presented. In them, the HADA aircraft is flying in level flight at a constant velocity, in helicopter mode with the wings folded under the fuselage (sweeping angle of 0 degrees). At a given time instant, wing unfolding is initiated at constant angular velocity, and it lasts 10 seconds. Sensor noise is considered as explained above and light to moderate atmospheric turbulence is also considered using the Dryden turbulence model [26,27].

**Figure 4.**
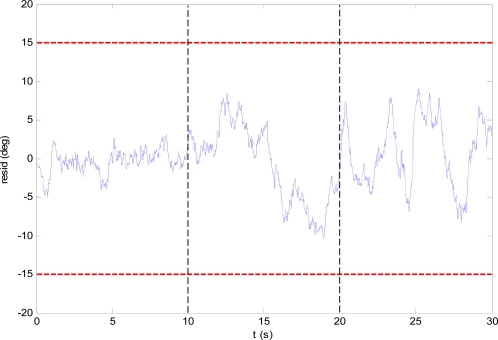
CUSUM filtered residual of GVIRT and GACT.

The residuals are obtained as the difference between the signals, and a CUSUM filter with forgetting factor is used to analyze them. Figure 4 shows the residual of the comparison between the virtual sensor angle estimate (GVIRT) and the wing angle derived from the actuator command (GACT), in fault-free conditions. The dashed vertical lines mark the initial and final times of the unfolding process. It can be seen that the turbulence and sensor noise cause variations in the residual, and that these variations are larger when the wings are unfolding and when they are unfolded than when the wings are under the fuselage. The red dashed horizontal lines denote the fault detection threshold level.

### 4.1. Sensor and Actuator Failure with Angle Sensor

This subsection presents an experiment with a failure in the wing angle sensor. The experiment is similar to the one described above: the HADA is in level flight in helicopter mode, and the wings are unfolded between *t* = 10 s and *t* = 20 s. The aircraft has an angle sensor connected to the wing unfolding mechanism. The sensor has had a hard failure, and it gives the same output regardless of the real wing sweeping angle. In this case it is straightforward to detect that there is a failure just comparing the angle sensor (GSENS) with the GACT signal (it is not shown here). From this comparison it will be detected that there has been a fault, but there is no information about where it is located.

**Figure 5.**
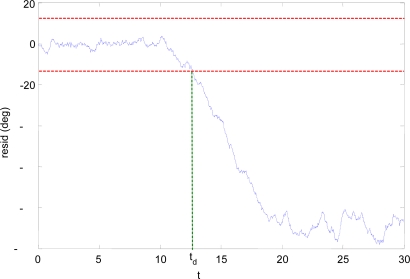
Angle sensor failure: CUSUM filtered residual of GVIRT and GSENS.

Figure 5 shows the results of the comparison between GSENS and the virtual sensor GVIRT signal. It can be seen that as the wings are unfolding, the residual begins to grow and clearly goes above the threshold level at *t* = *t_d_*, thus detecting that the fault is located at the angle sensor.

**Figure 6.**
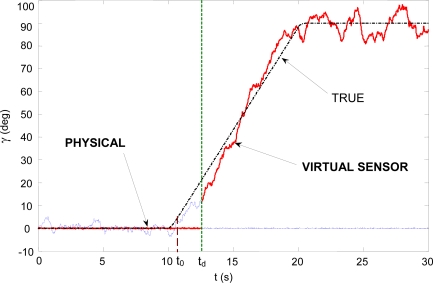
Wing sweeping angle estimation in case of sensor failure.

Moreover, it is possible to reconfigure the control system to use the angle virtual sensor once the fault has been detected and isolated. Figure 6 shows the “best” estimation of the wing sweeping angle: it is the angle sensor output when no fault is declared. At *t*_0_ a failure is detected (comparing GACT and GSENS), but the fault location is still unknown; at *t_d_* the fault is isolated and identified, and the virtual sensor reading is taken as the best estimate of the angle. Clearly the estimation of the virtual sensor is much noisier and less accurate, but it can give important information to the control system as to what action perform next.

In the same way, if the residual of GVIRT and GSENS do not grow larger that the threshold value it can be considered that the fault is located at the actuator and not the angle sensor.

### 4.2. Actuator Failure with No Angle Sensor Installed

**Figure 7.**
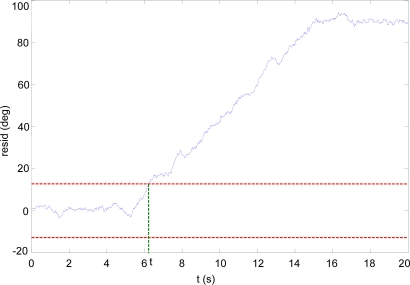
Actuator fault detection.

If there is no wing angle sensor installed, the virtual sensor can still be used to detect faults in the wing actuator. Figure 7 shows the residual generated by the comparison of the GACT and GVIRT signals when there is a fault in the wing actuator.

The actuator is instructed to unfold the wings at constant angular velocity between *t* = 5 s. and *t* = 15 s. It can be seen that the residual error begins to grow after the command, and goes above the threshold level, since the virtual sensor GVIRT detects that the actuator has not moved the wings but the GACT signal (reconstructed from the actuator command) “thinks” it has done so. In this case, it is also possible to use the virtual sensor as an anytime estimator of the wing sweeping angle.

## 5. Conclusions

Flight control in the transition phase of the HADA morphing aircraft is very important for mission success. When HADA is flying in helicopter mode and the wings are folding or unfolding, it is critical to know the real wing sweeping angle. This paper has shown how a virtual sensor can be used for FDIR of wing angle sensor and actuator failures. A nonlinear system identification technique has been used, since wing folding and unfolding is a complex nonlinear aerodynamic problem. The proposed method has been validated with experiments using a HADA simulator which includes real phenomena as sensor noise and sampling characteristics and turbulence and wind perturbations. The simulation experiments include detection of faults in the angle sensor and the angle actuator. In case of an angle sensor failure or if it is not available, the virtual sensor output can be used as a rough estimate of the wing sweeping angle, which gives valuable information to the HADA controller.
